# ABO‐Blood Group Associates With Survival Outcomes in Patients With Metastatic Non‐Small Cell Lung Cancer Treated With Pembrolizumab Monotherapy

**DOI:** 10.1111/1759-7714.70037

**Published:** 2025-03-20

**Authors:** Franziska Certa, Peter A. Horn, Julius Keyl, Bastian Mende, Smiths Lueong, Thomas Hilser, Sarah Theurer, Isabel Virchow, Yasmin Zaun, Michael Pogorzelski, Martin Metzenmacher, Halime Kalkavan, Stefan Kasper, Martin Schuler, Marcel Wiesweg, Gregor Zaun

**Affiliations:** ^1^ West German Cancer Center, Department of Medical Oncology University Hospital Essen Essen Germany; ^2^ Medical Faculty University Duisburg‐Essen Essen Germany; ^3^ Institute for Transfusion Medicine University Hospital of Essen Essen Germany; ^4^ West German Cancer Center, Institute of Pathology Essen University Hospital Essen Essen Germany; ^5^ Institute for Artificial Intelligence in Medicine University Hospital Essen Essen Germany; ^6^ Central Pharmacy University Hospital Essen Essen Germany; ^7^ German Cancer Consortium (DKTK) Partner Site University Hospital Essen Essen Germany; ^8^ West German Cancer Center, Institute for Developmental Cancer Therapeutics University Hospital Essen Essen Germany; ^9^ National Center for Tumor Diseases (NCT) West Essen Germany

**Keywords:** blood group, checkpoint inhibitor, non‐small cell lung cancer, predictive biomarkers

## Abstract

**Purpose:**

In patients with metastatic non‐small cell lung cancer (NSCLC) with high programmed death‐ligand 1 (PD‐L1) expression, there is still a lack of biomarkers to identify patients with maximum benefit from first‐line treatment with checkpoint inhibitor therapy (CIT) alone. This work examines the impact of different ABO blood groups (BG) on the response to CIT monotherapy.

**Methods:**

Retrospective analysis of patients with stage IV NSCLC and high PD‐L1 expression (tumor proportional score/TPS ≥ 50%), receiving first‐line therapy with pembrolizumab alone or in combination with chemotherapy at the West German Cancer Center from 2017 to 2022. Study endpoints were overall survival (OS) and progression‐free survival (PFS).

**Results:**

Eighty‐two patients were included in the analysis. Twenty‐two patients (27%) received first‐line therapy with pembrolizumab alone (monoimmunotherapy cohort/MIC), of which seven patients (32%) had BGO. Sixty patients (73%) were treated with pembrolizumab combined with platinum‐based chemotherapy (chemoimmunotherapy cohort/CIC), of which 38 (63%) had BGO. In MIC, younger age and BGO were independent predictors of favorable OS (BGO vs. other ABO‐BG: HR 0.22, 95% CI: 0.1–0.9; *p* = 0.037; median OS 62 versus 19 months) and PFS (BGO vs. other ABO‐BG: HR 0.21, 95% CI: 0.1–0.8; *p* = 0.024; median PFS 39 vs. 4 months). There was no significant impact of ABO‐BG in patients treated with CIC. In support, a historical control group treated with chemotherapy alone also showed no prognostic impact of the ABO‐BG.

**Conclusion:**

BGO associates with favorable survival in patients with NSCLC receiving pembrolizumab monotherapy, but not in patients with chemo‐immunotherapy or chemotherapy. Further validation of this promising strategy for personalized decision‐making is warranted.

## Graphical abstract

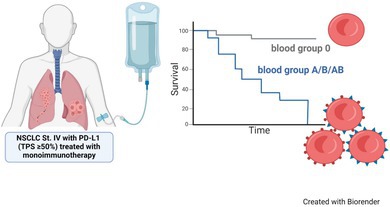

## 1 Introduction

Lung malignancies are the second most common cancer worldwide, with 2.2 million cases diagnosed and 1.7 million related deaths in 2020 [1]. About 80%–85% of all lung cancers are classified as non‐small cell lung cancer (NSCLC), with the two most common histological subtypes: Squamous cell carcinoma (20%–30%) and adenocarcinoma (40%–50%) [2, 3, 4]. About 50% of patients already have distant metastases (UICC stage IV [Union for international cancer control]) at first diagnosis. The late stage of initial diagnosis is partly responsible for the high mortality rate of the disease [5].

The treatment paradigm for lung cancer has changed in the last years: Nowadays, in particular for patients without targetable genetic alterations, checkpoint inhibitor therapy combined with platinum‐based chemotherapy is the standard of care in first‐line treatment in patients with metastatic NSCLC independently of PD‐L1 expression status [6, 7, 8]. In patients with high PD‐L1 expression (Tumor Proportion Score/TPS ≥ 50%) and absence of targetable genetic alterations, checkpoint inhibitor monotherapy, using atezolizumab, cemiplimab, or pembrolizumab, is also approved by the U.S. Food and Drug Administration (FDA) and European Medicines Agency (EMA) [9, 10, 11, 12, 13, 14, 15, 16]. Therefore, there are two approved first‐line treatment options for patients with metastatic NSCLC and high PD‐L1 expression.

Some analyses show a higher response rate to combination therapy in the first few months [17]. On the other hand, clinical practice also shows a higher rate of adverse side effects in these patients compared to immunotherapy alone [18, 19, 20]. However, there is still a lack of biomarkers to differentiate which patients with high PD‐L1 expression have the most likelihood of response to checkpoint inhibitor therapy (CIT) alone or which patient needs a combination of CIT and chemotherapy. Previous studies suggested a correlation between different blood groups and checkpoint inhibitor treatments in patients with various cancers: Chen et al. showed a correlation between the time to treatment failure and the patient's blood group. They described a benefit for patients with blood group O, but did not show survival data and had a very heterogeneous patient cohort with different tumor entities and treatment combinations [21].

In order to analyze a corresponding correlation in patients with metastatic NSCLC and high PD‐L1 expression and thereby improve the therapy situation in this patient group in the future, we investigated the impact of different blood groups on overall survival (OS) and progression‐free survival (PFS) in this distinct cohort.

## 2 Materials and Methods

### 2.1 Patient Selection

NSCLC patients treated with checkpoint inhibitors between December 2017 and April 2022 at the West German Cancer Center at the University Hospital Essen were analyzed. Subjects were included if they met predefined inclusion criteria: Available ABO blood group information, tumor entity NSCLC in stage IV, PD‐L1 expression TPS ≥ 50%, checkpoint inhibitor therapy as first‐line treatment with or without chemotherapy combination, and no other targeted treatment options in first line. Patients of the full analysis set (FAS) were further subgrouped into a chemo‐immunotherapy cohort (CIC) and a monoimmunotherapy cohort (MIC). In addition, a historical chemotherapy control cohort (CCC) was formed with patients who were treated with chemotherapy alone at the University Hospital Essen in the years 2015 and 2016 with NSCLC stage IV and who did not receive immunotherapy during the further course of treatment, with present ABO blood group information. Results on PD‐L1 expression of this control group were not available as this biomarker had not been established in standard of care diagnostics at that time.

**FIGURE 1:**
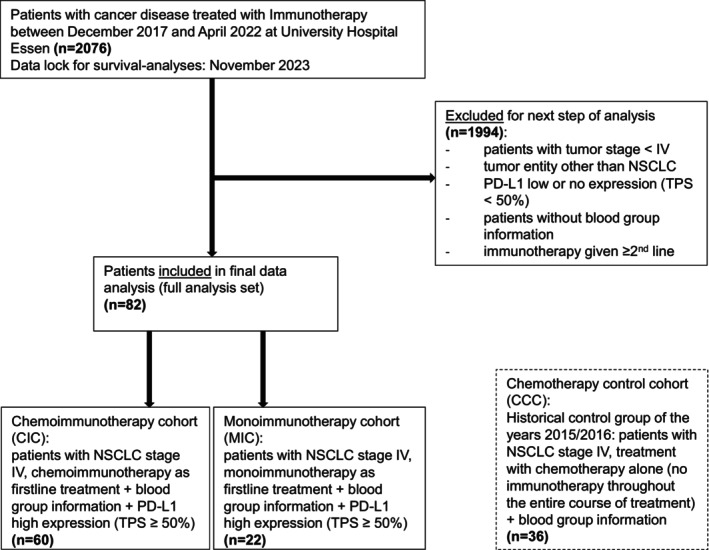
CONSORT (Consolidated Standards of Reporting Trials) flow diagram for patient inclusion in data analysis.

Data was retrieved from the electronic health records (EHR) of the University Hospital Essen. Data lock for survival analyses was November 2023. Figure 1 shows the CONSORT diagram. Due to a preliminary study that showed a correlation of immunotherapy efficacy with blood group O in NSCLC, due to the high prevalence of this blood group (e.g., half of the population in the USA has blood group O) [21] and due to the biological rationale investigating antigen‐bearing versus non‐antigen‐bearing blood group characteristics of the ABO blood group, we analyzed the outcome of patients with blood group O in comparison to patients with the remaining ABO blood groups (A, B and AB).

### 2.2 Statistical Analysis

Using Statistical Package for the Social Sciences (SPSS, version 29.0, IBM, Armonk, NY, USA) and MS Excel 2016 (version 2304, Microsoft, Richmond, WA, USA) pseudonymized data sets were analyzed. Overall survival (OS) and progression‐free survival (PFS) were analyzed using the Kaplan–Meier method and the log‐rank test. OS was defined as the time from the first diagnosis at tumor stage IV (UICC) to the date of death from any cause. PFS was defined as the time from therapy start to disease progression, the last documented visit at the University Hospital Essen, or death from any cause. For OS, patients alive or lost to follow‐up, and for PFS, patients without progression or lost to follow‐up at the data cutoff (November 2023) were censored at the last documented visit. Two‐sided *p*‐values < 0.05 were considered statistically significant. Prognostic parameters were assessed by multivariate analysis (Cox regression) of various reliably determinable clinical parameters, including different blood group information (ABO‐system), selected from the EHR for each patient. The study was approved by the Ethics Committee of the Medical Faculty of the University Duisburg‐Essen (Az‐22‐10 726‐BO).

## 3 Results

### 3.1 Patients

Two thousand and seventy‐six patients treated at the University Hospital Essen with checkpoint inhibitor therapy (CIT) between December 2017 and April 2022 were screened for the predefined inclusion criteria. Eighty‐two patients met these criteria and were included in this non‐randomized single‐center study (Full analysis set, FAS) (Figure 1). Of the FAS, 22 patients (27%) received a first‐line monoimmunotherapy (monoimmunotherapy cohort: MIC) and 60 patients (73%) a first‐line chemoimmunotherapy (chemoimmunotherapy cohort: CIC) (Figure 1). In the FAS, sex was balanced (50% female/male), median age at inclusion was 62 years, 45 patients (55%) had blood group O, and the most common histology was adenocarcinoma (*n* = 62, 82%).

The monoimmunotherapy cohort (MIC) contained more female patients (*n* = 14, 64%) and more patients ≥ 60 years (*n* = 16, 73%) versus 27 female patients (45%) and 36 patients ≥ 60 years (60%) in the chemoimmunotherapy cohort (CIC). Blood group O was detected in seven patients (32%) in the MIC and in 38 patients (63%) in the CIC. In terms of histological distribution, adenocarcinoma was dominant in both subgroups (MIC: *n* = 16, 73%; CIC: *n* = 51, 85%). All patients from the MIC were treated with pembrolizumab. In the CIC, the immunotherapy component was also pembrolizumab in all patients, while the chemotherapy backbone varied in some cases.

To explore the prognostic impact of the ABO‐BG, independent of immunotherapy, a control group (CCC; *n* = 36) of patients who were treated with chemotherapy alone (no immunotherapy at any treatment line) at the University Hospital Essen in the years 2015 and 2016 was analyzed. Results on PD‐L1 expression of this control group were not available as this biomarker had not been established in standard of care diagnostics at that time. In the CCC, 26 patients (72%) were male and 27 patients (75%) were younger than 60 years old. In 15 patients (42%) blood group O was detected. The dominant histological subtype was adenocarcinoma (*n* = 31; 86%). Table 1 provides an overview of the patient and tumor characteristics.

**TABLE 1:** Patient characteristics.

Parameter	Characteristics	Full analysis set (MIC + CIC) (*n* = 82)		Monoimmunotherapy cohort (MIC) (*n* = 22)		Chemoimmunotherapy cohort (CIC) (*n* = 60)		Historical chemotherapy control cohort (CCC) (*n* = 36)	
		*n*	%	*n*	%	*n*	%	*n*	%
Sex	Male	41	50	8	36	33	55	26	72
	Female	41	50	14	64	27	45	10	28
Age	≥ 60 years	52	63	16	73	36	60	9	25
	< 60 years	30	37	6	27	24	40	27	75
ABO‐System	O	45	55	7	32	38	63	15	42
	Other	37	45	15	68	22	37	21	58
	A	25	30	11	50	14	24	13	36
	B	8	10	3	14	5	8	6	17
	AB	4	5	1	4	3	5	2	5
Histology	Adenocarcinoma	67	82	16	73	51	85	31	86
	Other	15	18	6	27	9	15	5	14
	Undifferentiated carcinoma	4	5	2	9	2	3		
	Squamous cell carcinoma	10	12	3	14	7	12	5	14
	Neuroendocrine carcinoma	1	1	1	4	0	0		
Affected organ systems	1	34	41	6	27	28	47	19	53
	> 1	48	59	16	73	32	53	17	47
Firstline therapy	Pembrolizumab			22	100				
	Cisplatin/Pemetrexed/Pembrolizumab					29	48		
	Cisplatin/Paclitaxel/Pembrolizumab					4	7		
	Carboplatin/Paclitaxel/Pembrolizumab					14	23		
	Carboplatin/Pemetrexed/Pembrolizumab					13	22		
	Cisplatin							2	6
	Carboplatin							4	11
	Gemcitabine							1	2
	Cisplatin/Paclitaxel							8	22
	Cisplatin/Pemetrexed							6	17
	Carboplatin/Paclitaxel							3	8
	Carboplatin/Pemetrexed							8	22

	Carboplatin/Gemcitabine							2	6
Carboplatin/Vinorelbin							2	6

*Note:* Median age was 62 years (range, 40–84 years).

### 3.2 Survival and Prognostic Factors of the Full Analysis Set (FAS)

The median OS in the FAS was 17.0 months [95% CI: 7.4–26.6] (Supporting Information 1a) and PFS was 9.0 months [95% CI 7.2–10.8] (Supporting Information 1b). The median OS in the MIC (*n* = 22) was 24.0 months (95% CI 12.5–35.5) compared with 15.0 months (95% CI 10.0–20.0) in the CIC (*n* = 60) (Supporting Information 1a). PFS was 10.0 months (95% CI 0.0–24.9) in the MIC versus 9.0 months (95% CI 7.9–10.1) in the CIC (Supporting Information 1b). Nevertheless, there was no significant difference in either OS (*p* = 0.743) or PFS (*p* = 0.26). However, both Kaplan–Meier curves of OS and PFS showed an early decline in the monoimmunotherapy curve, which disappeared over time.

In multivariate Cox‐regression analysis of the FAS, only younger age (< 60 years) was an independent prognostic factor for favorable OS (*p* = 0.037; HR 0.534, 95% CI 0.297–0.962) (Supporting Information 2a) and PFS (*p* = 0.017; HR 0.522, 95% CI 0.307–0.889) (Supporting Information 2b).

### 3.3 Survival and Prognostic Factors of Both Subgroups (MIC and CIC) and the Chemotherapy Control Cohort (CCC)

**FIGURE 2:**
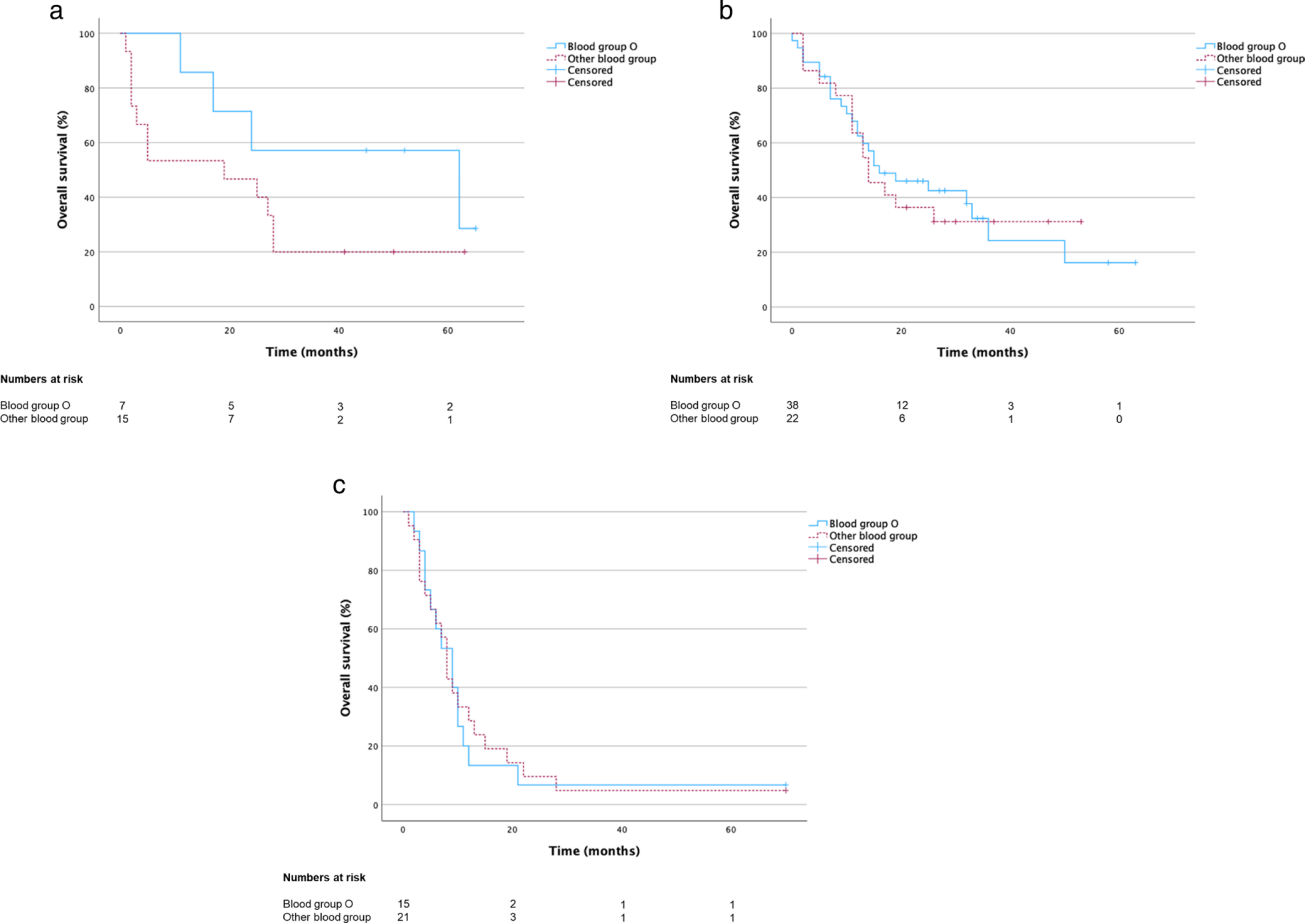
Median overall survival (OS) of patients with NSCLC stage IV (a) PD‐L1 high expression and monoimmunotherapy (MIC; *n* = 22) (b) PD‐L1 high expression and chemoimmunotherapy (CIC; *n* = 60) (c) historical chemotherapy control cohort (CCC; *n* = 36) (PD‐L1 expression unknown).

**FIGURE 3:**
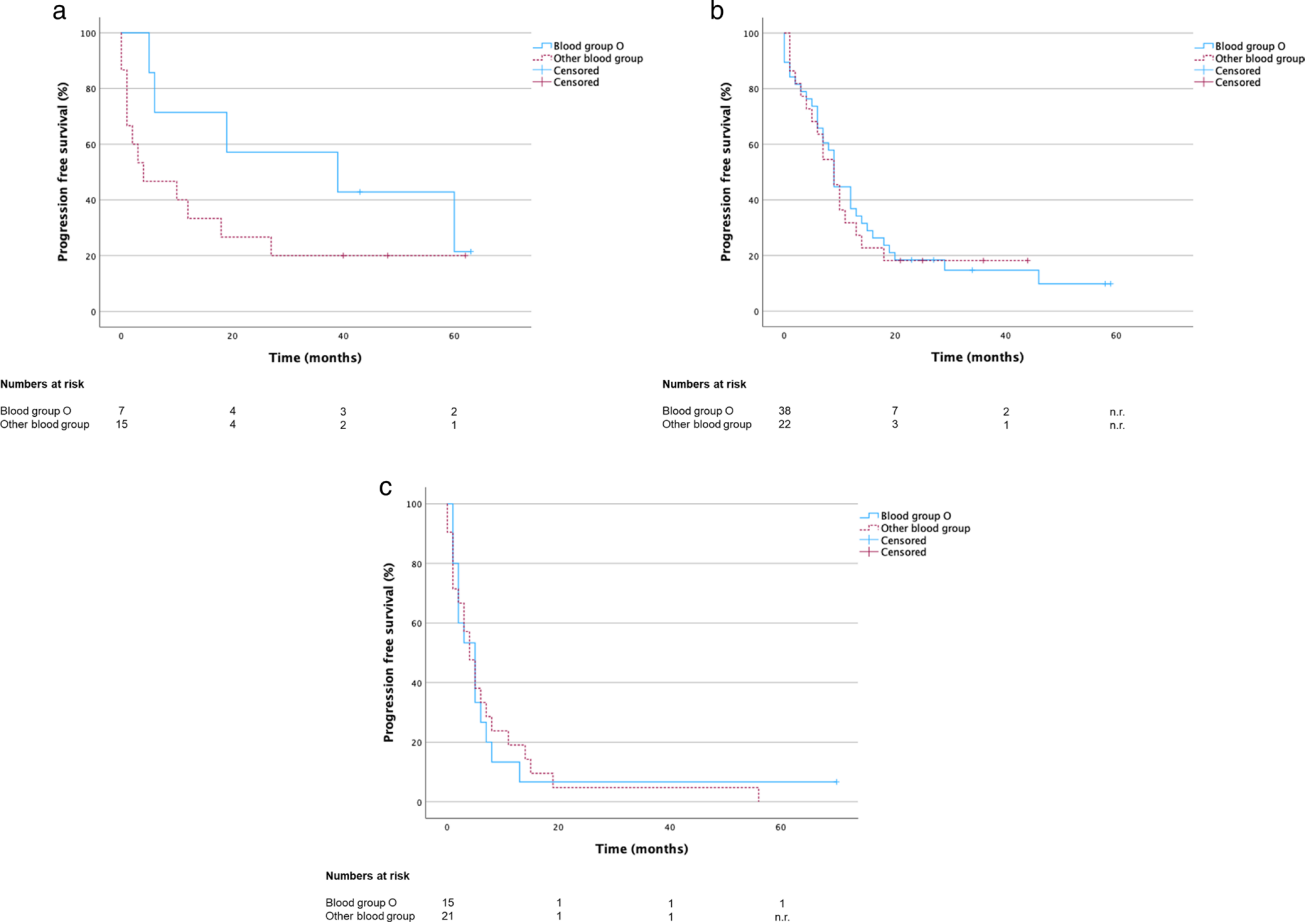
Progression‐free survival (PFS) of patients with NSCLC stage IV (a) PD‐L1 high expression and monoimmunotherapy (MIC; *n* = 22) (b) PD‐L1 high expression and chemoimmunotherapy (CIC; *n* = 60) (c) historical chemotherapy control cohort (CCC; *n* = 36) (PD‐L1 expression unknown).

Next, we aimed to identify prognostic factors to estimate whether a patient could benefit sufficiently from immunotherapy alone or whether a combination of chemotherapy and immunotherapy would be more favorable. To address this, we first analyzed different clinical parameters to examine their prognostic value using the univariate Kaplan–Meier method: We could show a difference in both OS and PFS in the MIC regarding the ABO blood groups. The median OS of patients with blood group O in the MIC was 62 months (95% CI 4.0–120.0) versus 19.0 months (95% CI 0.0–39.8) for patients with other ABO blood groups (Figure 2a, Table S1) and the PFS was 39.0 months (95% CI 0.0–90.3) versus 4.0 (95% CI 0.0–14.1) respectively (Figure 3a, Table S1). However, the difference was not significant in the univariate Kaplan‐Meier analysis (OS: *p* = 0.18; PFS: *p* = 0.187). In contrast, in the CIC, we could not show a trend between the ABO blood groups: The median OS of patients with blood group O in the CIC was 16.0 months (95% CI 3.8–28.2) versus 14.0 months (95% CI 8.5–19.5) for patients with other ABO blood groups (Figure 2b, Table S1) and the PFS was 9.0 months in both groups (BG O: 95% CI 7.8–10.2; other ABO‐BG: 95% CI 5.9–12.1) (Figure 3b, Table S1) (OS: *p* = 0.853; PFS: *p* = 0.859).

To determine whether blood group O has no prognostic effect when receiving chemotherapy compared to MIC, we formed a historical chemotherapy control cohort (CCC; *n* = 36) treated in the years 2015 and 2016, who did not yet receive immunotherapy at any time: With an OS of 8.0 months [95% CI 6.0–10.0] and a PFS of 4.0 months [95% CI 2.3–5.7] (Supporting Information 1c and 1d) survival data in the CCC group were clearly worse than in the MIC and CIC. But, in line with the data from the CIC, there was no difference in OS and PFS in the CCC with regard to the ABO‐blood group (OS: BG O (*n* = 15) 9.0 months [95% CI 5.3–12.7] vs. other ABO‐BG (*n* = 21) 8.0 months [95% CI 6.5–9.5]; *p* = 0.863; Figure 2c) (PFS: BG O 5.0 months [95% CI 2.3–7.7] vs. other ABO‐BG 4.0 months [95% CI 1.8–6.2]; *p* = 0.988; Figure 3c).

**FIGURE 4:**
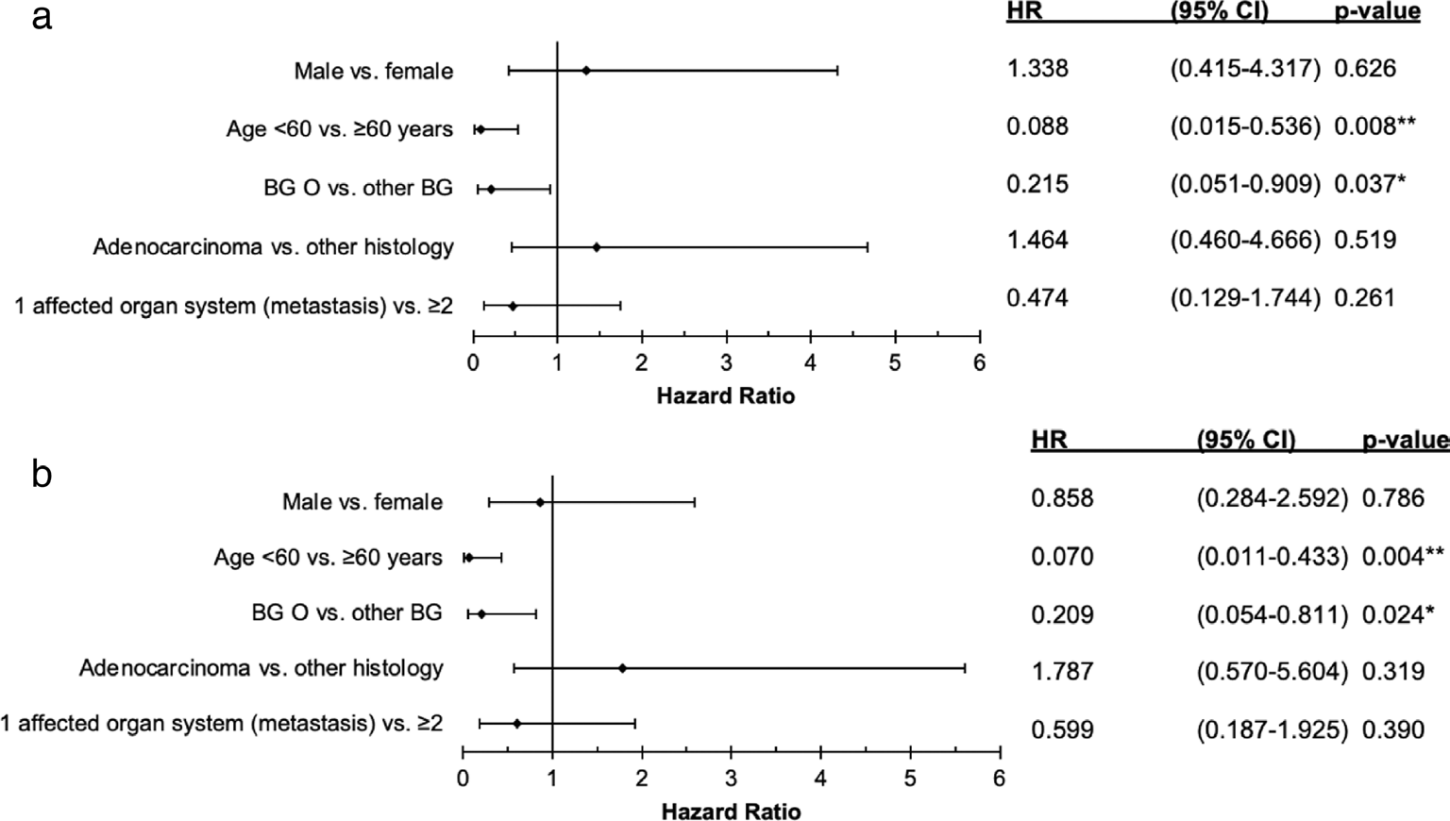
Multivariate analysis (Cox regression) for prognostic parameters in different subgroups (HR: Hazard ratio with 95% CI and P value. *p < 0.05. **p < 0.01. BG = blood group) (a) monoimmunotherapy cohort (MIC; *n* = 22): In relation to Overall survival (OS) (b) monoimmunotherapy cohort (MIC) in relation to Progression‐free survival (PFS).

Due to the apparent, albeit not statistically significant difference in survival related to blood groups in the MIC in the univariate analysis and no difference in both chemotherapy associated groups (CIC and CCC), we next performed a multivariate analysis (Cox regression) of the clinical parameters to eliminate possible confounding factors: In the multivariate analysis within the MIC, besides young age (Hazard ratio [HR] 0.09, 95% CI 0.0–0.5; *p* = 0.008), we identified blood group O as an additional favorable prognostic marker for OS with a HR of 0.22 (95% CI 0.1–0.9) and a *p*‐value of 0.037 compared to other blood groups (Figure 4a). In addition, both prognostic favorable markers were also confirmed for PFS in the MIC: younger age < 60 years had a HR of 0.07 (95% CI 0.0–0.4; *p* = 0.004) and blood group O a HR of 0.21 (95% CI 0.1–0.8; *p* = 0.024) (Figure 4b).

Interestingly, in the CIC and the CCC, the analyzed parameters were not prognostic, especially not the blood group (Supporting Information 3a–d).

## 4 Discussion

The aim of this study was to detect routinely determinable prognostic parameters that allow a better stratification of patients and improved therapy allocation in a selected patient cohort with metastatic NSCLC and high PD‐L1 expression without targetable genetic alterations in the first‐line situation.

In the monoimmunotherapy cohort (MIC), we identified younger age and blood group O as significant prognostic favorable parameters regarding OS and PFS. In contrast, in the two chemotherapy‐associated control groups (chemoimmunotherapy/CIC and chemotherapy control cohort/CCC) of this study, no significant impact of the blood group could be observed.

With a median OS of 62 months for patients with blood group O compared to only 19 months for other ABO‐blood groups (PFS: BG O: 39 months versus other ABO‐BG: 4 months), our study demonstrates that immunotherapy alone could be sufficient for patients with metastatic NSCLC with high PD‐L1 expression and blood group O. Interestingly, an OS and PFS benefit was lacking in the ABO‐blood groups in the chemoimmune cohort (CIC) and the historical chemotherapy control cohort/CCC.

Consequently, our data strongly suggest that chemoimmune combination therapy should always be considered in patients with other ABO‐blood groups than blood group O in patients with metastatic NSCLC and PD‐L1 high expression. Furthermore, the prognostically favorable effect of blood group O in our study could be used as a parameter for patient stratification in future immunotherapy trials. Because of the retrospective nature of our study, there are some differences between the analyzed subgroups: In addition to the lower number of patients in the MIC, blood group O is only represented in around one‐third of the patients in the MIC, compared to around two‐third in the CIC.

With 42% patients with BG O, the historical control group CCC lies between MIC and CIC. These differences could only be eliminated by prospective studies in future. Regardless of the retrospectively partly differently composed control groups, the detection of a prognostically favorable effect of blood group O in the monoimmune therapy cohort is still valid through the multivariate analysis, the stringently formed subgroups by predefined inclusion criteria and by using two different control groups, which, unlike the monoimmunotherapy group, showed no prognostic effect of the blood group.

The literature has so far shown different results regarding the correlation between blood group antigens and the response to immunotherapy in different tumors: Ergun et al. examined the treatment response of nivolumab in patients with advanced malignant melanoma. They found a significantly higher OS and PFS in patients with blood group B. Another study examined a correlation between blood group and the response to immunotherapy in advanced renal cell carcinoma (RCC) in the second, third, and fourth‐line situation after tyrosine kinase inhibitor therapy. No significant differences were found in either survival or multivariate Cox regression [22]. In contrast to our study, in addition to different tumor entities, patients with higher treatment lines were also included in these studies.

The data from Chen et al. support our work: They were also able to show a prognostically favorable effect of blood group O in patients treated with immunotherapy. However, they focused on the time to treatment failure of immunotherapy in comparison of different blood groups and analyzed a highly heterogeneous patient group with different tumor entities as well as different treatment modalities that were not evaluated separately using immunotherapy alone, double immunotherapy, and chemoimmune combination. Approximately 21% of their analyzed patients had NSCLCs, and 41% of the total population had blood group O [21].

To avoid these possible confounding factors such as different tumor entities or treatment lines and different PD‐L1 expressions, in our work we used a clearly defined population with regard to the tumor entity NSCLC with high PD‐L1 expression of the tumor and evaluated the different therapy modalities with chemoimmunotherapy combination and monoimmunotherapy separately. In addition, only patients with immunotherapy in the first‐line setting were included, and we added a historical chemotherapy control cohort to verify our results. Furthermore, we focused on survival data and were able to identify blood group O in the monoimmunotherapy subgroup as a significant prognostically favorable parameter with regard to OS and PFS in the multivariate analysis. Due to the relatively small number of patients in the strictly defined subgroups, the univariate analyzes using Kaplan–Meier method showed a relatively higher OS and PFS in the monoimmune subgroup with blood group O, but no significant median OS and PFS data compared to the significant data in the multivariate analysis with blood group O as a prognostic parameter.

In summary, our retrospective data show blood group O in patients with metastatic NSCLC with PD‐L1 high expression as a significantly favorable prognostic parameter for a monoimmunotherapy. Future immunotherapy studies should prospectively validate these retrospective results in a larger patient population to investigate whether patients with blood group O in the aforementioned constellation benefit sufficiently from a monoimmunotherapy and patients with other blood group antigens of the ABO‐system should receive a chemoimmunotherapy combination.

## 5 Author Contributions

**Franziska Certa:** conceptualization; data curation; formal analysis; investigation; methodology; software; visualization; writing – original draft; and writing – review and editing. **Peter A. Horn:** data curation; investigation; supervision; writing – review and editing. **Julius Keyl:** software; investigation; writing – review and editing. **Bastian Mende:** software; investigation; writing – review and editing. **Smiths Lueong:** investigation; writing – review and editing. **Thomas Hilser:** investigation; writing – review and editing. **Sarah Theurer:** investigation; writing – review and editing. **Isabel Virchow:** investigation; writing – review and editing. **Yasmin Zaun:** investigation; writing – review and editing. **Michael Pogorzelski:** investigation; writing – review and editing. **Martin Metzenmacher:** investigation; writing – review and editing. **Halime Kalkavan:** investigation; writing – review and editing. **Stefan Kasper:** conceptualization; investigation; methodology; supervision; writing – review and editing. **Martin Schuler:** conceptualization; data curation; methodology; supervision; validation; writing – review and editing. **Marcel Wiesweg:** conceptualization; methodology; supervision; validation; writing – review and editing. **Gregor Zaun:** conceptualization; data curation; formal analysis; investigation; methodology; project administration; software; supervision; validation; visualization; writing – original draft; and writing – review and editing.

## Ethics Statement

This retrospective study was approved by the Ethics Committee of the Medical Faculty of the University of Duisburg‐Essen (Az‐22‐10 726‐BO).

## Conflicts of Interest

The authors declare no conflicts of interest. Outside the submitted work the following authors declare their potential conflicts of interest: Thomas Hilser received Honoraria for consultancy from IPSEN Pharma. Stefan Kasper received honoraria from Merck Serono, MSD, Novartis, BMS, Amgen, Roche, Sanofi‐Aventis, Servier, Incyte and Lilly such as research funding from Merck Serono, Lilly, BMS and Roche. Martin Metzenmacher received honoraria for advisory boards from Amgen, Astra Zeneca, Johnson&Johnson, MSD, Novartis, Novocure, Pfizer and Takeda. Michael Pogorzelski received honoraria for consultancy from Amgen, Boehringer Ingelheim, BMS, GSK, Merck Healthcare KGaA, MSD and Takeda. Martin Schuler has received research funding for his institution from AstraZeneca and Bristol Myers‐Squibb and Janssen. He has received honorarium for CME presentations from Amgen, Bristol‐Myers Squibb, GSK, Janssen, MSD, Roche, Sanofi and Novartis. He had a compensated consultant role for Amgen, AstraZeneca, Boehringer Ingelheim, Bristol‐Myers Squibb, GSK, Janssen, Merck Serono, MSD, Novartis, Roche, Sanofi and Takeda. Marcel Wiesweg received honoraria for advisory role from Amgen, AstraZeneca, Bristol‐Myers Squibb, Daiichi Sankyo, GSK, Janssen, Novartis, Pfizer, Roche and Takeda. He received research funding from Bristol‐Myers Squibb and Takeda and travel support from Amgen, Daiichi Sankyo and Janssen.

## Supporting information


**DATA S1.** Supporting Information.

## Data Availability

The datasets generated and analyzed during the current study are available from the corresponding author on reasonable request.
