# Supplementary material for: ABO‐Blood Group Associates With Survival Outcomes in Patients With Metastatic Non‐Small Cell Lung Cancer Treated With Pembrolizumab Monotherapy
Source: Thorac Cancer. 2025 Mar 20;16(6):e70037. doi: 10.1111/1759-7714.70037 (PMC11925720; doi:10.1111/1759-7714.70037)
Supplement: Supplementary file 1 — DATA S1. Supporting Information. [file TCA-16-e70037-s001.docx]

**Supplementary material**

**Blood group associates with survival outcomes in patients with metastatic non-small cell lung cancer treated with pembrolizumab monotherapy**

Franziska Certa^a,h^, Peter A. Horn^b,h^, Julius Keyl^f,h,i^, Bastian Mende^c,h^, Smiths Lueong^d,e,h^, Thomas Hilser^a,h^, Sarah Theurer^f,h^, Isabel Virchow^a,h^, Yasmin Zaun^a,h^, Michael Pogorzelski^a,h^, Martin Metzenmacher^a,h^, Halime Kalkavan^a,d,g,h^, Stefan Kasper^a,g,h^, Martin Schuler^a,g,h^, Marcel Wiesweg^a,g,h^, Gregor Zaun^a,h^**^#^**

^a^ West German Cancer Center, Department of Medical Oncology, University Hospital Essen, Essen, Germany

^b^ Institute for Transfusion Medicine, University Hospital of Essen, Essen, Germany

^c^ Central Pharmacy, University Hospital Essen, Essen, Germany.

^d^ German Cancer Consortium (DKTK), Partner site University Hospital Essen, Essen, Germany

^e^ West German Cancer Center, Institute for Developmental Cancer Therapeutics, University Hospital Essen, Essen, Germany

^f^ West German Cancer Center, Institute of Pathology Essen, University Hospital Essen, Germany

^g^ National Center for Tumor Diseases (NCT) West, Essen, Germany

^h^ Medical Faculty, University Duisburg-Essen, Essen, Germany

^i^ Institute for Artificial Intelligence in Medicine, University Hospital Essen, Essen, Germany

**#Correspondence:**

Dr. Gregor Zaun, Email: gregor.zaun@uk‑essen.de, Tel.: +49-(0)201 723 2011,

Fax: +49-(0)201 723 5747

***
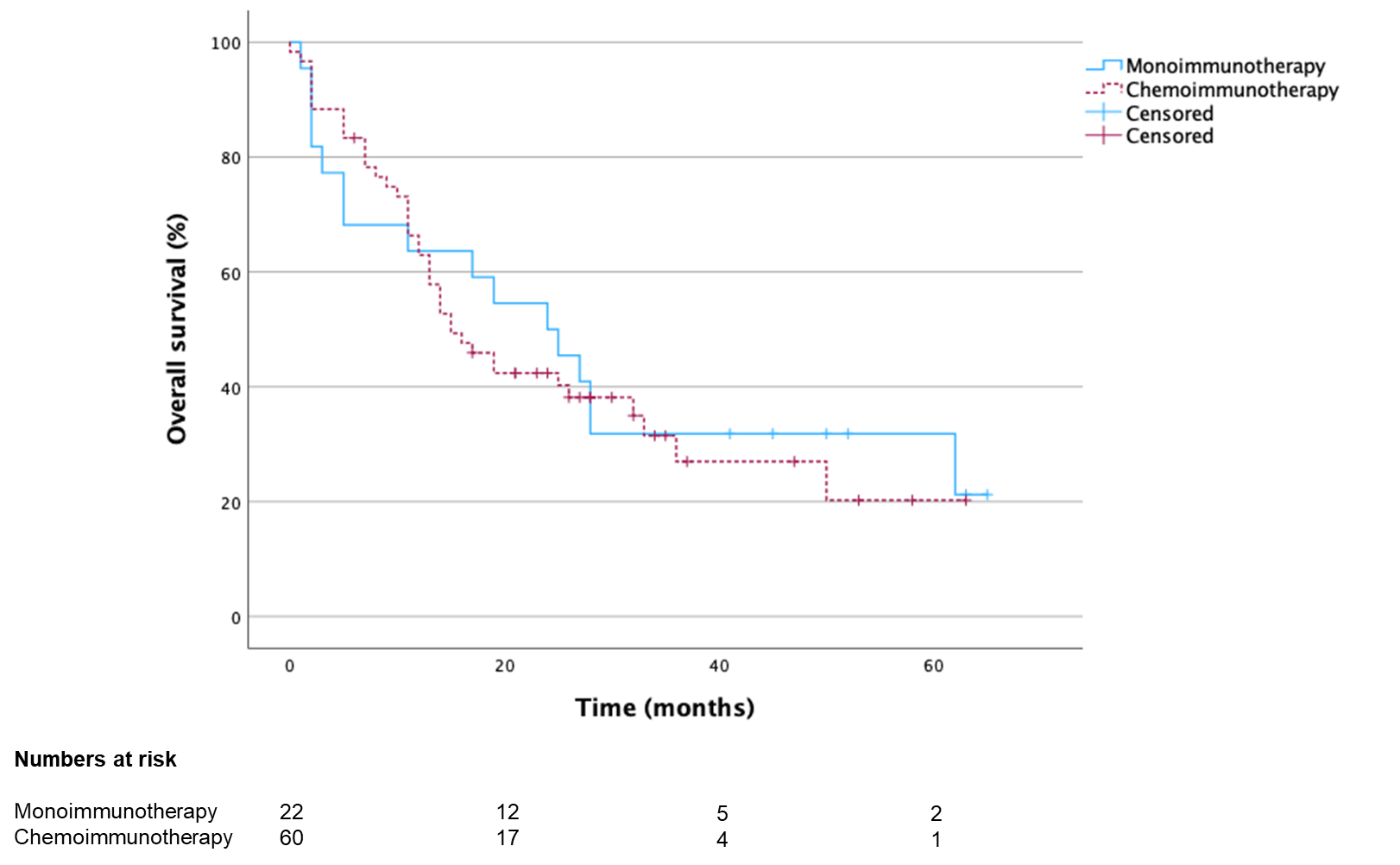
***

***Suppl. 1a*** *Median overall survival (OS) of patients with NSCLC stage IV and PD-L1 high expression: OS of full analysis set (MIC + CIC; n=82) was 17.0 months [95% CI 7.4 – 26.6]. OS of monoimmunotherapy (MIC; n=22) was 24.0 months [95% CI 12.5 – 35.5] vs. chemoimmunotherapy (CIC; n=60) 15.0 months [95% CI 10.0 – 20.0]; p = 0.74*


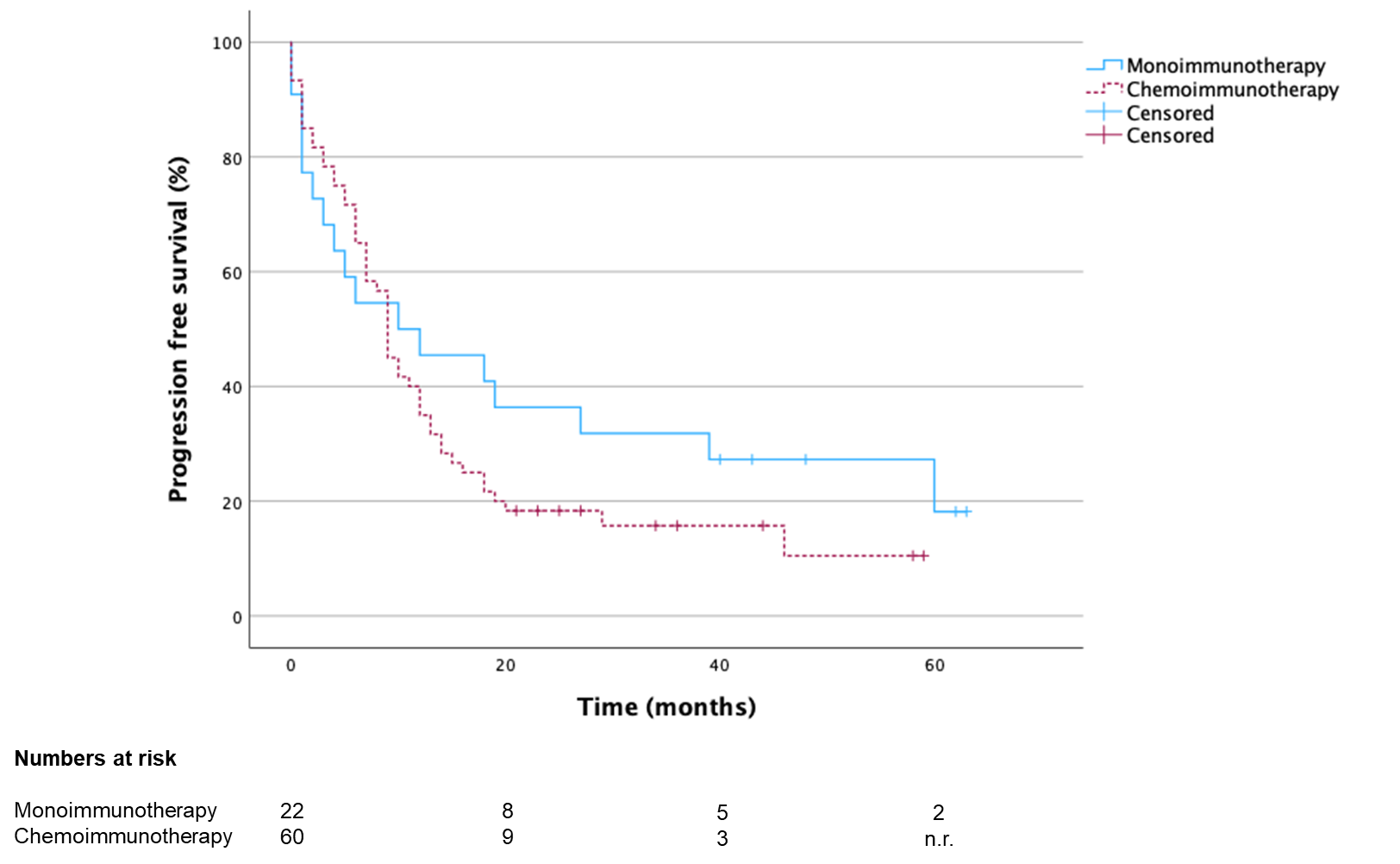


***Suppl. 1b*** *Progression-free survival (PFS) of patients with NSCLC stage IV and PD-L1 high expression: PFS of full analysis set (MIC + CIC; n=82) was 9.0 months [95% CI 7.2 – 10.8]. PFS of monoimmunotherapy (MIC; n=22) was 10.0 months [95% CI 0.0 – 24.9] vs. chemoimmunotherapy (CIC; n=60) 9.0 months [95% CI 7,9 – 10.1]; p = 0.26*


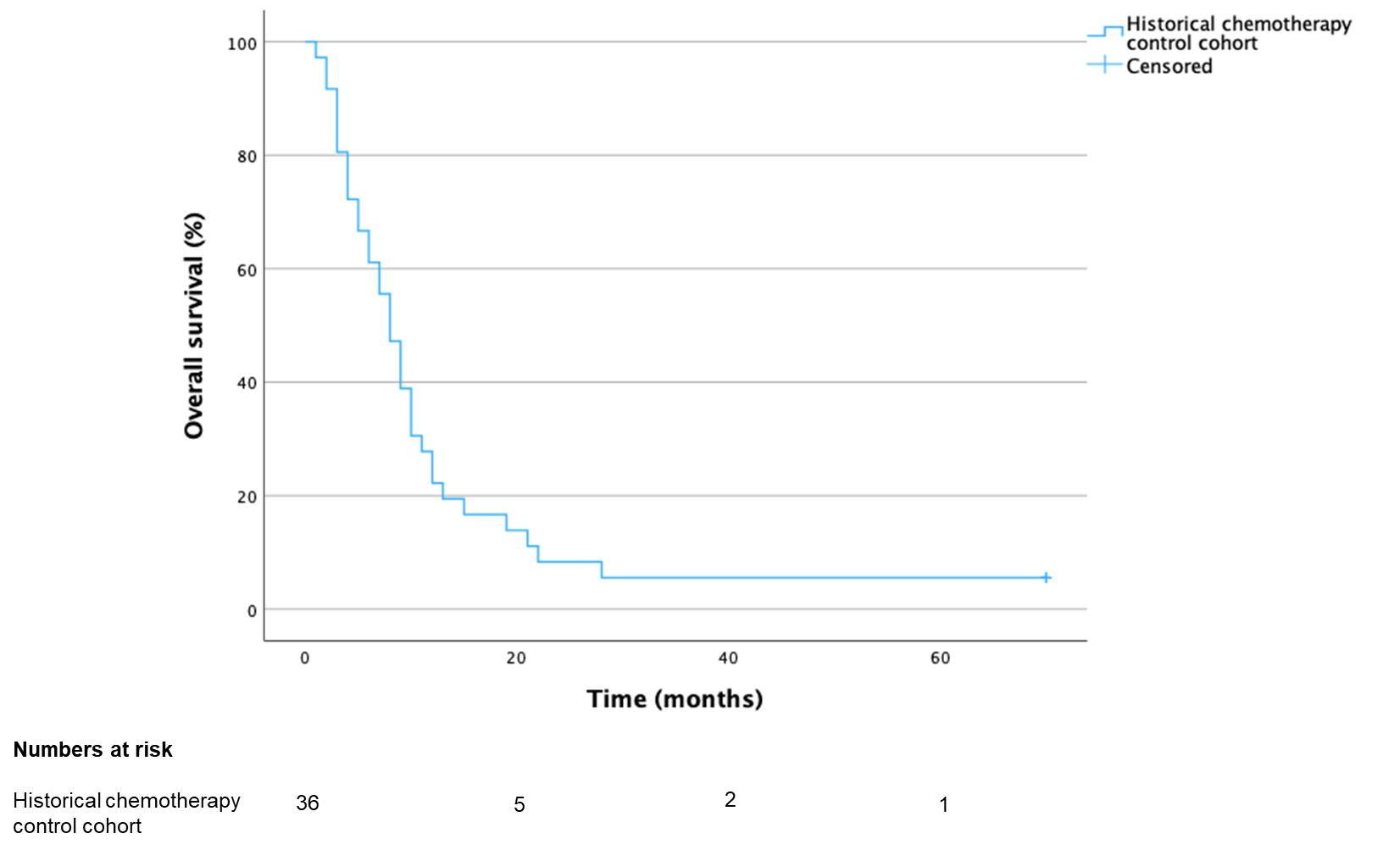


***Suppl. 1c*** *Median overall survival (OS) of patients with NSCLC stage IV of the historical chemotherapy control group (CCC; n=36) (PD-L1 expression unknown) 8.0 months [95% CI 6.0 – 10.0]*


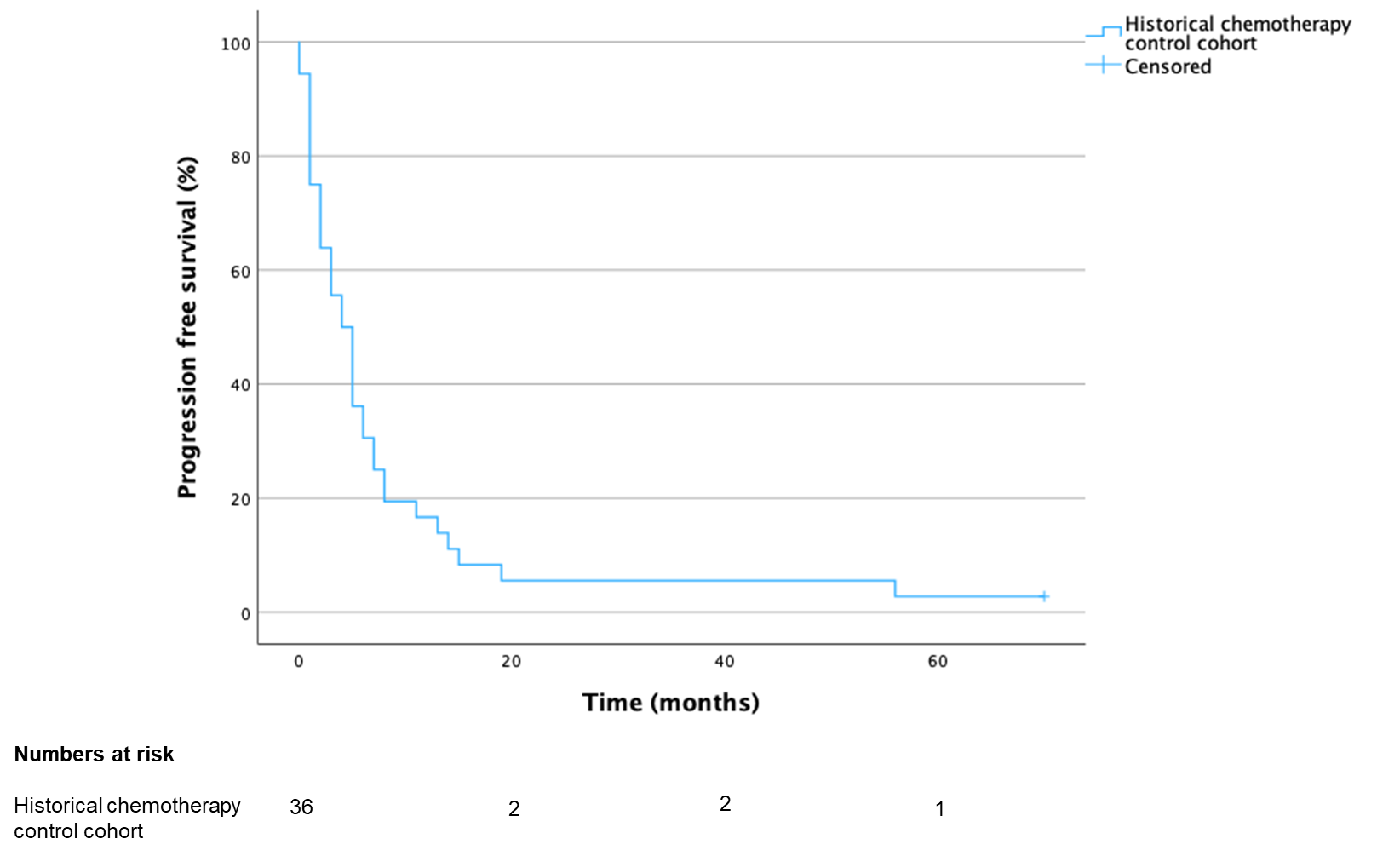


***Suppl. 1d*** *Progression-free survival (PFS) of patients with NSCLC stage IV of the historical chemotherapy control group (CCC; n=36) (PD-L1 expression unknown) 4.0 months [95% CI 2.3 – 5.7]*

**Suppl. 2a**


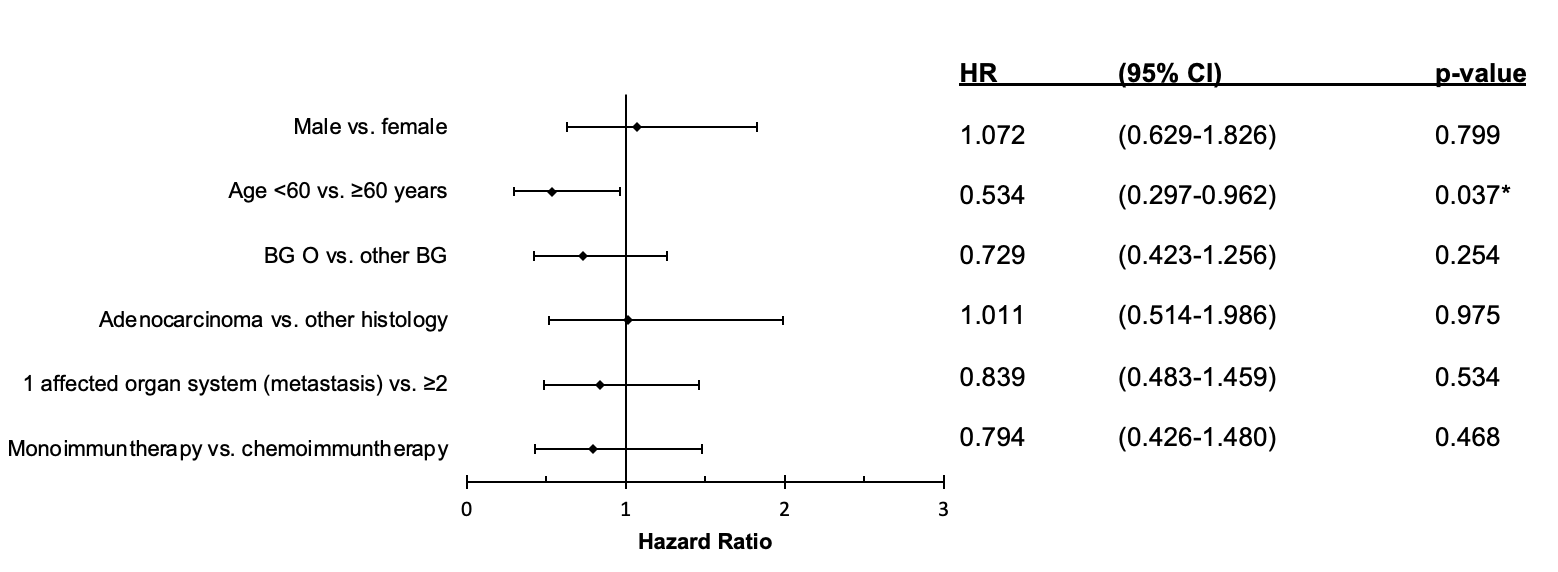


**Suppl. 2b**


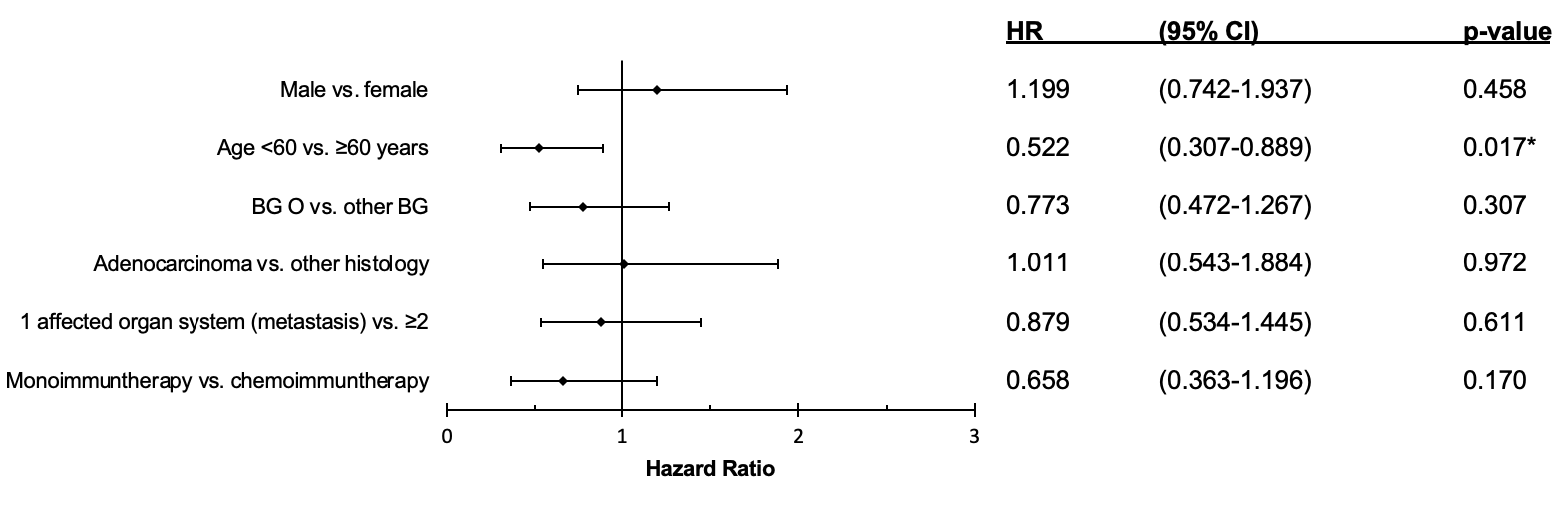


***Suppl. 2*** *Multivariate analysis (Cox regression) for prognostic parameters for full analysis set with NSCLC (n=82) (hazard ratio/HR with 95% CI and P value. *P < 0.05. BG = blood group.* ***a.*** *in relation to Overall survival (OS)* ***b.*** *in relation* **to** *Progression-free survival (PFS)*

**Suppl. 3a**


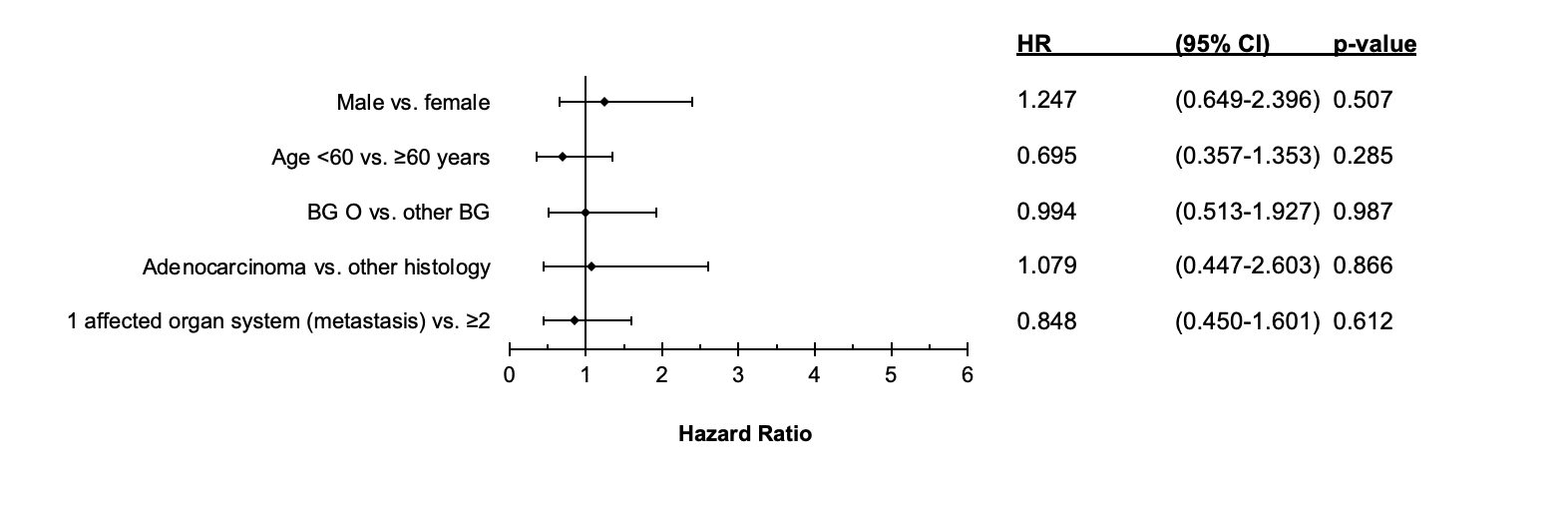


**Suppl. 3b**


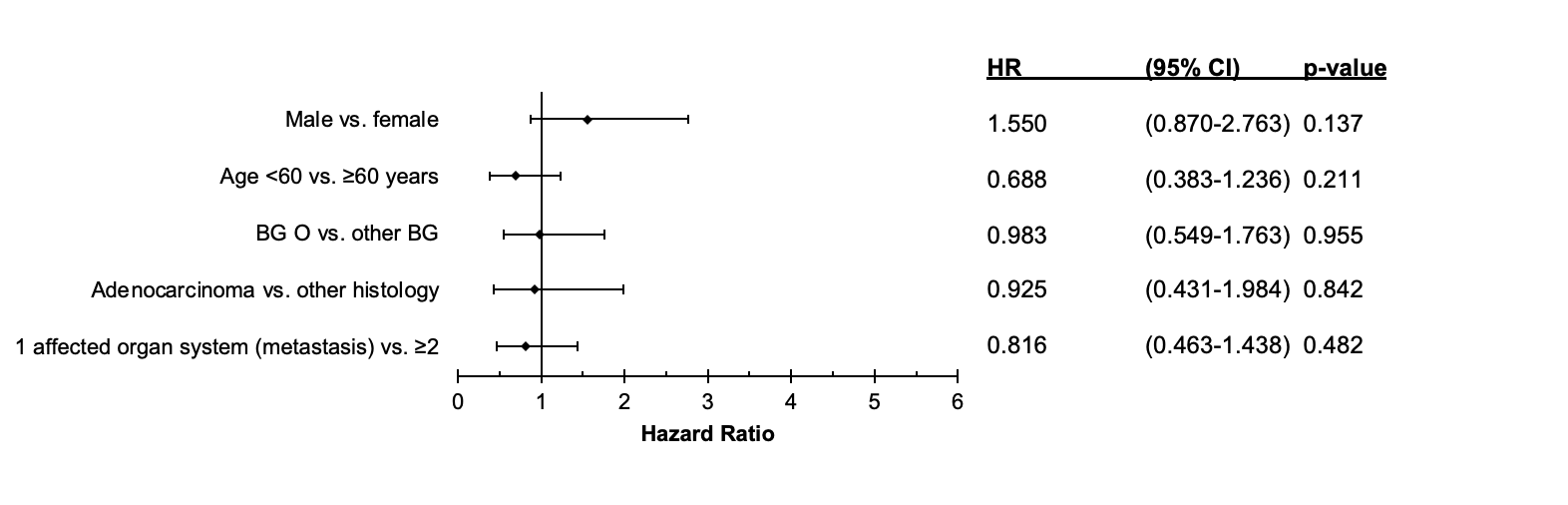


**Suppl. 3c**


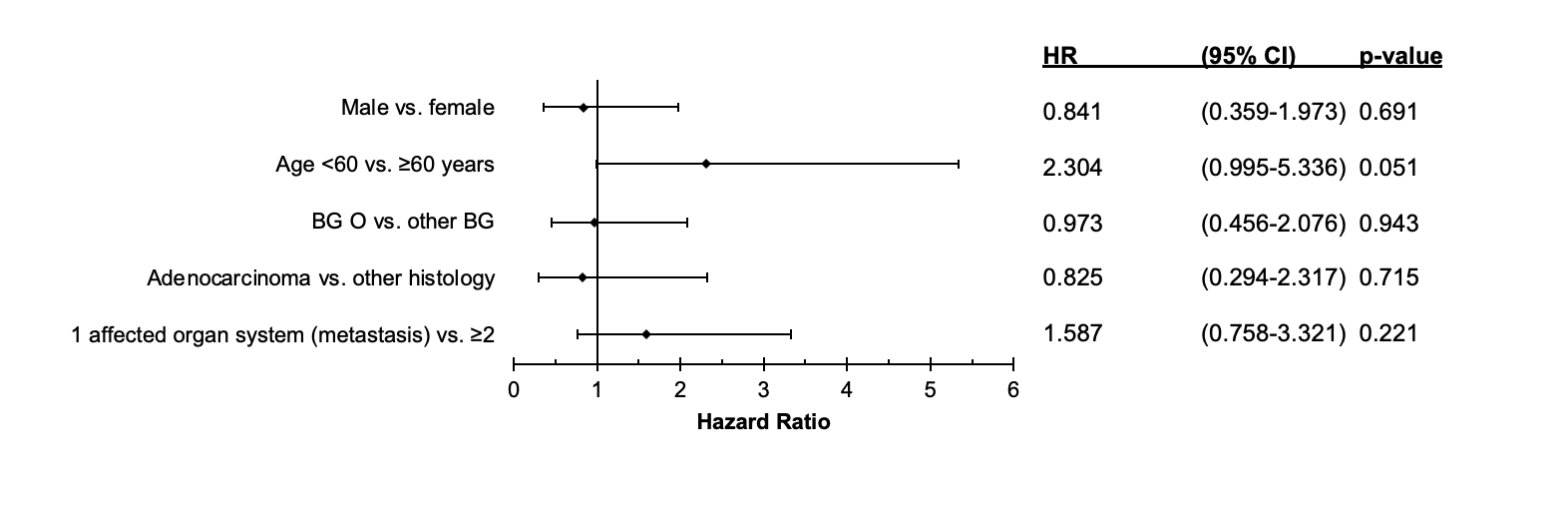


**Suppl. 3d**


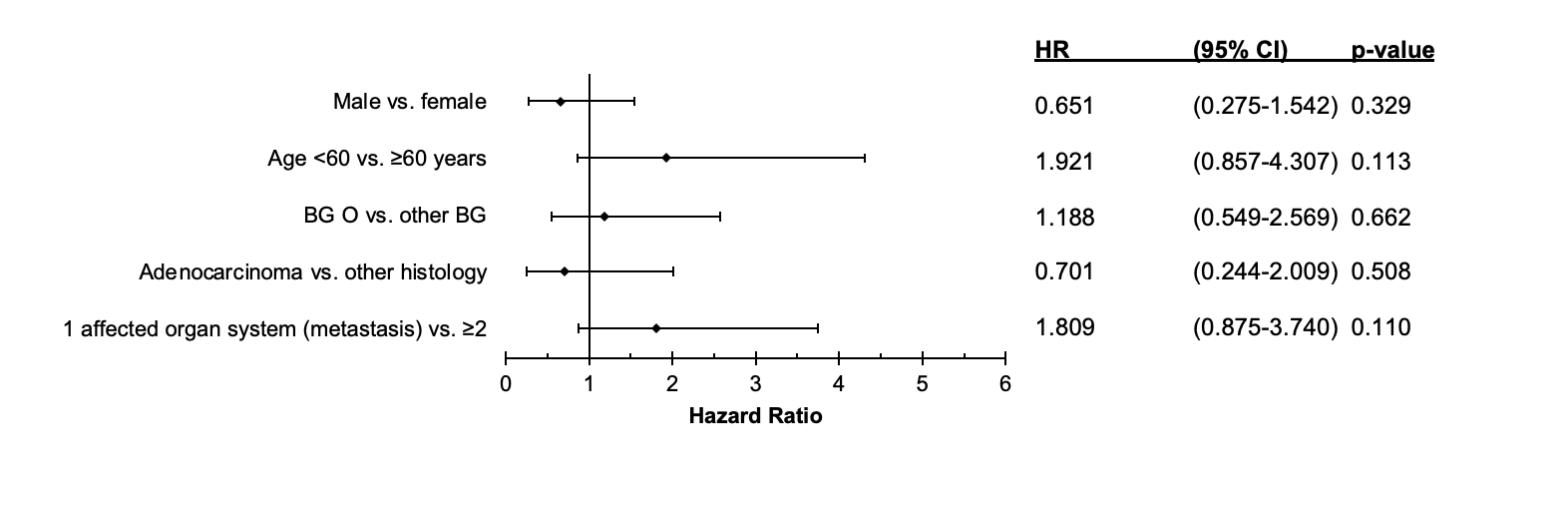


**Suppl. 3** Multivariate analysis (Cox regression) for prognostic parameters in different subgroups (HR: hazard ratio with 95% CI and P value. BG = blood group: **a.** chemoimmunotherapy cohort (CIC; n=60): in relation to Overall survival (OS) **b.** chemoimmunotherapy cohort (CIC): in relation to Progression-free survival (PFS) **c.** historical chemotherapy control cohort (CCC; n=36): in relation to Overall survival (OS) **d.** historical chemotherapy control cohort (CCC): in relation to Progression-free survival (PFS)

***Suppl. table 1*** *Comparison of overall survival (OS) and progression-free survival (PFS) according to the blood group*
